# Supplementary material for: Fungal Endophytes Isolated from Elymus repens, a Wild Relative of Barley, Have Potential for Biological Control of Fusarium culmorum and Pyrenophora teres in Barley
Source: Pathogens. 2022 Sep 25;11(10):1097. doi: 10.3390/pathogens11101097 (PMC9612256; doi:10.3390/pathogens11101097)
Supplement: Supplementary file 1 [file pathogens-11-01097-s001.zip › pathogens-1908759-supplementary.pdf]

Table S1. Overview of the inoculation of seeds with endophytes.

Pathogen	Experiment	Endophyte spore suspension (1:2 w/v)	Endophyte spore suspension (1:2 w/v)	10 min soaking at 130 rpm	24 h soaking at 130 rpm	Dried 30 min in laminar air flow cabinet	Dried for 2 hours in laminar air flow cabinet	Dried for 12 hours in laminar air flow cabinet
Fusarium culmorum	**1**	X		X		X		
	**2**	X		X		X		
	**3**	X		X		X		
	**4**	X		X		X		
	**5**		X	X		X		
	**6**	X		X				X
Pyrenophora teres	**7**	X		X		X		
	**8**	X		X		X		
	**9**	X			X		X	

Method used for *Agrobacterium* mediated transformation of *Periconia macrospinosa* strain E1 using GFP

The *Periconia macrospinosa* strain E1 was transformed using the *A. tumefaciens* strain AGL1 containing the GFP transformation plasmid pPZP201-GG-BH. The promoter region of the *Blumeria graminis gpd* (glyceraldehyde-3-phosphate dehydrogenase) gene regulates the *gfp* gene and the *B. graminis* β-tubulin promoter region regulates the *hph* gene encoding hygromycin B resistance. Fungal tolerance to hygromycin B was tested on PDA supplemented with hygromycin B in the following concentrations 0, 50, 100, 150, 200, 250 and 300 µg/mL. Bacteria containing the GFP plasmid were grown on LB plates with half salt concentration and 0.1 mg/mL ampicillin, 0.05 mg/mL kanamycin and 0.05 mg/mL rifampicin for 2 days at room temperature. *Agrobacterium* cultures were transferred into an autoclaved glass tube with screw cap containing 10 mL half salt LB media and 0.1 mg/mL ampicillin, 0.05 mg/mL kanamycin and 0.05 mg/mL rifampicin, and incubated tilted for 24 h at 28 °C at 220 rpm. A culture solution OD660 of 0.45 was measured in a 1:10 (v/v) dilution with MiliQ water and the standard blank reference used was diluted LB medium with half salt concentration and 0.1 mg/mL ampicillin, 0.05 mg/mL kanamycin and 0.05 mg/mL rifampicin. 5 mL *Agrobacterium* culture was spun down at 1449 x *g* for 3 min. The pellet was resuspended in 10 mL induction medium (IM: composition, see **Error! Reference source not found.**2) with 200 µM acetosyringone and incubated for 3 h at 28 °C.

Spores from endophytes were harvested in IM media and spore suspensions were filtered through cheesecloth. The spore concentration was adjusted to 2×10^6 spores/mL. Four sterile filter papers (Black round 7.5 cm diameter filters, Frisinette Aps) were cut into 16 pieces. Four pieces were placed on each plate of CM agar (same as IM, but instead of 10 mM glucose, 5 mM glucose and 15 g Bacto agar was used) supplemented with 200 µM acetosyringone. Equal volumes of *Agrobacterium* and fungal spore solutions were mixed. A volume of 25 µL of the mix was pipetted out on to the individual filters and spread over them with a spatula. Plates were incubated at 25 °C for two days with the lid side up. Individual filters were transferred to Czapek Dox agar supplemented with 100 µg/mL hygromycin B and 95 µg/mL cefoxitin. Plates were incubated at 25 °C for 4-8 days. Fungal cultures that grew successfully from the filters into the medium were selected. A volume of 10-15 µL MiliQ water was pipetted up and down on the part of the fungal culture growing on the medium. The spore suspension was spread with a Drigalski spatula on PDA plates containing 100 µg/mL hygromycin B and 95 µg/mL cefoxitin. Plates were left to incubate at 25 °C for 2-3 days. Individual germinating spores were transferred to PDA with 100 µg/mL hygromycin B.

Confirmation of the transformation

The transformation was checked under a fluorescence microscope (Olympus BX60) using excitation filters for GFP (excitation 455-495 nm, dicroitic mirror DM 505, barrier filter 510-555 nm). Seven individual transformants were prepared and plugs stored in 10 % glycerol at -80 °C. Three replications were made of the wild type and the seven transformants and their growth was measured at three time points, at 5, 7 and 9 days. Furthermore, the intensity of their fluorescence emission was evaluated on a scale from 1-5 (Figure S1).

Table S2. Composition of 1000 mL induction medium (IM) + acetosyringone

Volume	Concentration
10 mL	1M K_2HPO_4
10 mL	1M KH_2PO_4
2.5 mL	1M NaCl
4 mL	1M $(NH_4)_2SO_4$
10 mL	50% (w/v) Glycerol
910 mL	MilliQ water
Autoclave for 20 min at 120 ˚C	
1 mL	9mM $FeSO_4$ (filter sterile)
10 mL	1M Glucose (autoclaved)
40 mL	1M MES (pH=5.3; filter sterile*)
	* make freeze aliquots of 40 mL
1.4 mL	0.5M $CaCl_2$ (autoclaved)
2 mL	1M $MgSO_4$
200 μM acetosyringone = 20 mL 10 mM AS	

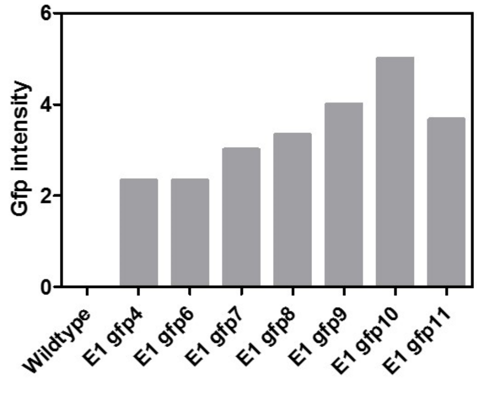

Figure S1. The GFP intensity of seven gfp strains and the wild type evaluated on a subjective scale from 0-5. Each column represents the mean of three Petri dishes. No statistical evaluation was made because of the subjective nature of the assessment.

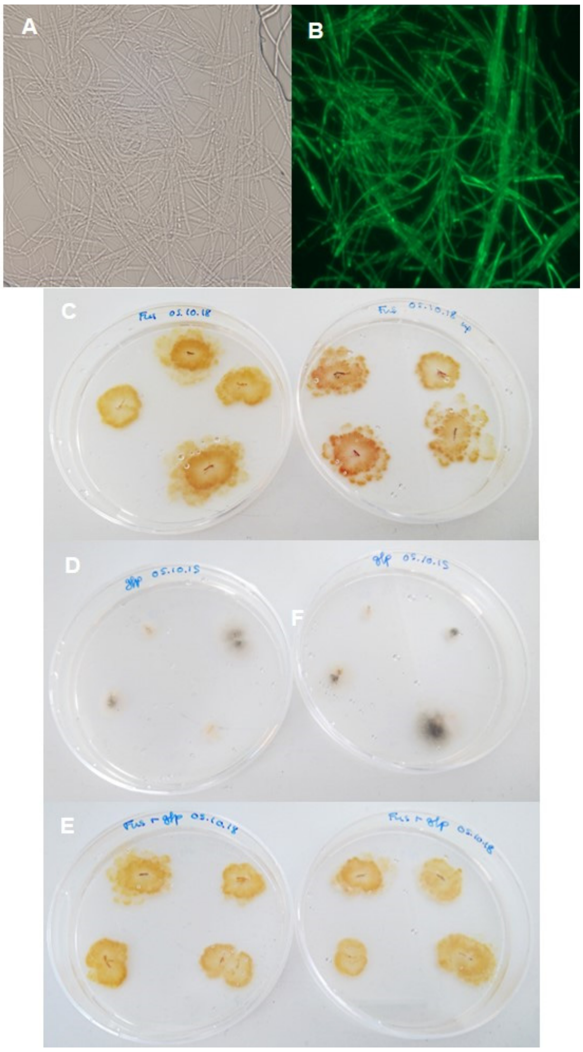

Figure S2. Confirmation of transformation of E1gfp10 (A-B) and growth from washed root segments at 14 days after sowing from experiment 6 (C-E). A) Hyphae of E1gfp10 under normal light, B) Hyphae of E1gfp10 under fluorescent light, C-E) Growth of mycelium from four root segments per Petri dish. Root pieces were taken from 14 days old plants from experiment 6. C) *Fusarium culmorum* treated plants. D) E1gfp10 treated plants and E) *Fusarium culmorum* and E1gfp10 treated plants. Green fluorescence of hyphae growing from root pieces confirmed presence of E1gfp10f on roots of seed treated plants.
